# PAK5-mediated AIF phosphorylation inhibits its nuclear translocation and promotes breast cancer tumorigenesis

**DOI:** 10.7150/ijbs.58102

**Published:** 2021-03-27

**Authors:** Yao Xing, Yang Li, Bingtao Hu, Fuyi Han, Xin Zhao, Hongyan Zhang, Yanshu Li, Danni Li, Jiabin Li, Feng Jin, Feng Li

**Affiliations:** 1 Department of Cell Biology, Key Laboratory of Cell Biology of National Health Commission of the PRC, Key Laboratory of Medical Cell Biology of Ministry of Education of the PRC, China Medical University, No.77, Puhe Road, Shenyang, 110122, Liaoning, China.; 2 Department of Breast Surgery, Department of Surgical Oncology, Research Unit of General Surgery, The First Affiliated Hospital of China Medical University, No. 155, North Nanjing Street, Heping District, 110001 Shenyang, Liaoning, China.; 3 Department of Medical Oncology, The First Affiliated Hospital of China Medical University, Shenyang, China.

**Keywords:** PAK5, AIF, breast cancer, apoptosis

## Abstract

Although p21 activated kinase 5 (PAK5) is related to the progression of multiple cancers, its biological function in breast cancer remains unclear. Apoptosis-inducing factor (AIF) is a vital apoptosis factor in mitochondria, which can be released from mitochondria and enter the nucleus, causing caspase-independent apoptosis. In this study, we reveal that PAK5 inhibits apoptosis by preventing the nuclear translocation of AIF. PAK5 inhibits the release of AIF from mitochondria in breast cancer cells by decreasing the mitochondria membrane permeability and increasing the membrane potential. Furthermore, PAK5 phosphorylates AIF at Thr281 site to inhibit the formation of AIF/importin α3 complex, leading to decrease AIF nuclear translocation. Functionally, we demonstrate that PAK5-mediated AIF phosphorylation promotes the proliferation of breast cancer cells and accelerates the growth of breast cancer *in vivo*. Significantly, PAK5 and AIF expression in breast cancer are positively correlated with poor patient prognosis. PAK5 expression is negatively correlated with AIF nuclear translocation. These results suggest that PAK5-AIF signaling pathway may play an essential role in mammary tumorigenesis, providing a new therapeutic target for the treatment of breast cancer.

## Introduction

Globally, breast cancer is the most common cause of cancer diagnosis and death in women, accounting for about a quarter of cancer cases in women 1. In the past decades, through the progress of modern surgery technology and the innovation of chemotherapy drugs, the prevention and treatment of breast cancer has made significant progress 2. However, for advanced breast cancer, the prognosis of patients is still inferior, and the median overall survival (OS) is approximately 24-30 months 3-5. Therefore, it is particularly important to explore the molecular mechanism and therapeutic targets for breast cancer.

P21 activated kinase 5 (PAK5) is recently discovered as a member of the PAK family and plays an essential role in cell survival and cytoskeleton stability 6. Increasing evidence shows that PAK5 is an oncogenic protein, which is overexpressed in multiple cancers and contributes to the progression of tumor 7, 8. PAK5 has been reported to regulate cyclin D1 to promote G1/S transition of the cell cycle, which promotes cell proliferation 9. PAK5 can phosphorylate serine 161 and 187 site of GATA1 to recruit HDAC3/4 to inhibit E-cadherin transcription, leading to EMT in breast cancer cells 10. PAK5-mediated NF-κB-p65 phosphorylation promoted breast cancer cell proliferation 11. In addition, PAK5 has been shown to phosphorylate S119 site of DNPEP to facilitate its ubiquitination, which promotes the proliferation and metastasis of breast cancer 12. Although these studies have shown that PAK5 is a crucial kinase in breast cancer progression, its potential molecular mechanism has not been fully elucidated.

Mitochondria are the key factors in the initiation and execution of apoptosis 13, 14. When stimulated by a series of external signals, mitochondria releases apoptosis-inducing factors such as AIF, endonuclease G and Cytochrome c into the cytoplasm, and activating caspase-dependent or caspase-independent pathways to induce apoptosis 15, 16. AIF is anchored to the inner membrane of mitochondria and performs the function of oxidoreductase under normal physiological conditions. When the cells are stimulated by apoptosis signal, calpain I and one or more cathepsins located in mitochondria cleave AIF, making it a 57kDa soluble Pro-apoptotic protein AIF (mature AIF). AIF is released from mitochondria into the cytoplasm and enters the nucleus, which binds with DNA to cause chromatin condensation and large-scale DNA degradation to induce caspase-independent apoptosis 17-19. The release of AIF from mitochondria to nuclear is regulated by many factors 20, 21. However, the effect of PAK5 on AIF function has not been reported, and the clinical impact of AIF on breast cancer progression is still not clear.

In this study, we found that PAK5 could prevent the release of AIF from mitochondria and inhibit apoptosis by phosphorylation of AIF. Importantly, our research showed that with the development of breast cancer, AIF nuclear translocation was significantly reduced, which was closely related to the high expression of PAK5. These results suggested that PAK5-AIF signaling pathway might be a new potential therapeutic target for the treatment of breast cancer.

## Materials and methods

### Cell culture

HEK-293 and MDA-MB-231 breast cancer cells were cultured in DMEM; BT474 breast cancer cells were cultured in RPMI-1640 medium. All cells were supplemented with 10% FBS and purchased from Chinese Academy of Sciences (Shanghai, China).

### Plasmid and lentiviral vector construction and transfection

Myc-PAK5, GFP-PAK5, GST-importins and GST-PAK5 were generated in our laboratory. Flag-AIF 67 kDa (WT and T281A mutant), Flag-AIF 57 kDa (WT, S202A mutant and T281A mutant), His-AIF (67 kDa, 62 kDa, 57 kDa), GST-AIF (WT and T281A mutant) and GST-truncated proteins of AIF were constructed by using PCR amplification. The indicated cells were transfected with the appropriate plasmids using Higene (Applygen, Beijing, China). PAK5-Lentivirus was purchased from GeneChem Company (Shanghai, China). 1 × 10^5^ cells were infected with 2 μl of 1 × 10^9^ TU lentivirus for 24 hours and selected with 2 μg/ml puromycin.

### Western blot, immunoprecipitation and GST pull-down assay

Immunoprecipitation, western blot analyses and GST pull-down assay have been described previously 22.

### Cell/Tissue Immunofluorescence

Cell/Tissues were blocked with goat serum, and incubated with the primary antibody overnight at 4 °C and subsequently incubated with secondary antibody conjugated with green or red dye for 40 minutes. Nucleus was stained using DAPI (Roche Applied Science) for 15 minutes. Fluorescence microscope (Nikon, Tokyo, Japan) was used to acquire images.

### Antibodies and reagents

Antibodies against proteins were used in the experiments in follows: AIF and LaminB1 (Abcam, Cambridge, MA, #ab32516, #16048); PAK5 (R&D Systems, Minneapolis, MN, #MAB4696); Myc (Santa Cruz Biotechnology, Dallas, TX, # 9E10); β-Tubulin, GFP and His (GenScript, Nanjing, China, #2G7D4, # SD1223); GAPDH (protein tech, Wuhan China, # 60004-1-Ig); Flag (Sigma, St Louis, MO, # F1804); Bcl2, Bax and Phospho-Serine R-X-Y/F-X-pS Motif (Cell Signaling Technology [CST], Danvers, MA, #4223, #2774, #9614).

### Patient tissues and specimens

122 cases of human breast cancer tissues and matched adjacent normal tissues were collected from the First Hospital of China Medical University. Patient clinical information was investigated and recorded, and all samples signed the patients' informed consent form. The histological grade of breast cancer was assessed according to the World Health Organization.

### Colony formation assay

1 × 10^3^ Cells were cultured in 6-well plates until visible cell colonies were formed. The cells are fixed and stained, and the number of colonies was measured.

### CCK8 assay

CCK8 reagent was added to incubate at 37 °C for 2 h, and the laser detection with the wavelength of 450 nm was used. The data was calculated according to the reagent instructions.

### Tumor xenograft analysis

The 2 × 10^6^ stably transfected BT474 cells were injected subcutaneously into the female nude mice (nu/nu, 4-5 weeks old, Vital River Laboratories, Beijing, China). Tumor volume was measured once every 3 days. The calculation formula was tumor volume= π/6 × (L × W × H). Mice were killed after 30 days. The work was carried out in strict accordance with the Animal Ethics Committee of China Medical University.

### Immunohistochemistry

Immunohistochemistry has been described previously 12. 75 cases of breast cancer paraffin sections were collected between January 2008 to July 2009 from the First Hospital of China Medical University. 10 cases of Para cancer, 50 cases of breast benign tumor and 50 cases of breast cancer were collected between July 2018 to July 2019 from the First Hospital of China Medical University.

### Apoptosis assay and Mitochondria Membrane Potential (Δψm)

Cells were digested and cultured in 6-well plates. The cells were fixed following the protocol from the Annexin V-FITC Apoptosis Detection Kit and the Mitochondrial membrane potential assay kit with JC-1 (Beyotime, ShangHai, China).

### Cytoplasmic and nuclear protein extraction

Cytoplasmic and nuclear proteins were extracted by using the Nuclear and Cytoplasmic Protein Extraction Kit (Beyotime, ShangHai, China).

### Mitochondrial protein extraction

The mitochondrial proteins were extracted by using the Mitochondrial Isolation Kit for Cultured Cells (APPLYGEN, Beijing, China).

### Protein phosphorylation mass spectrometry

GST-AIF were purified *in vitro* and washed with kinase buffer (25 mM Tris, pH 7.5). Active PAK5 Kinase (PV4441, Life Technologies Corp, New York, USA) was used for PAK5 kinase assay in 50 μl of kinase buffer containing 200 uM of ATP for 30 min at 30 °C. The reaction was terminated by boiling water bath for 10 minutes and detected by mass spectrometry.

### Statistical analysis

We used GraphPad Prism software for statistical analyses. Student's t-test was applied to compare the groups' differences. The significance of differences in clinicopathological data was determined with a chi-square test. *P* < 0.05 was considered statistically significant. The Kaplan-Meier method was used to generate survival curves.

## Results

### PAK5 and AIF expression positively correlated with poor patient prognosis

**Figure 1:**
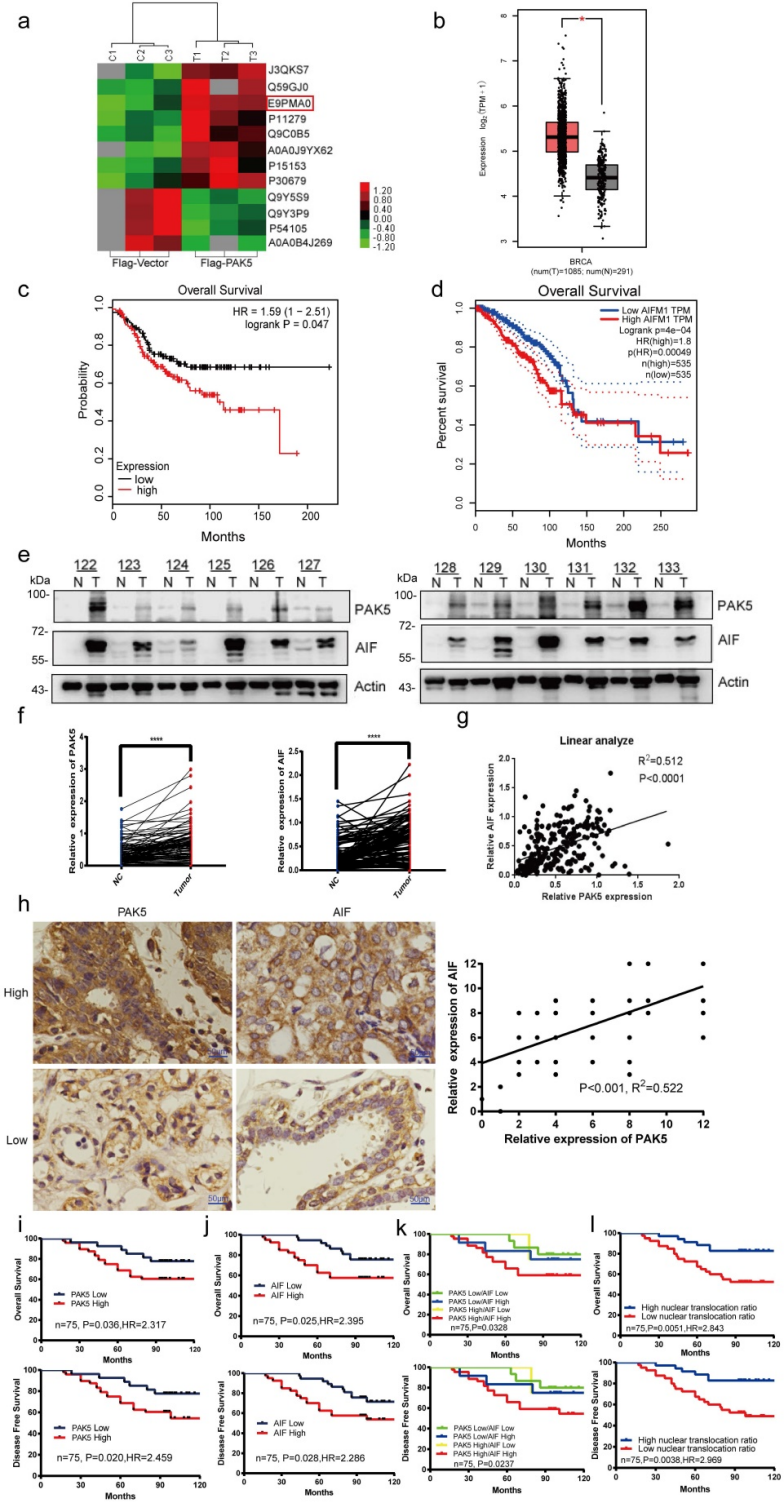
** PAK5 and AIF expression positively correlated with poor prognosis of breast cancer.** (a) Analysis of expression differential proteins regulated by PAK5. Lysates from MDA-MB-231 cells overexpressing Flag-vector or Flag-tagged PAK5 and screening of differentially expressed proteins by sequencing. (b) Predict the expression of AIF in breast cancer and adjacent cancer in TCGA database. The fold-change was 0.897. (c) Predict the overall survival of AIF in GSE31519 database. (d) Predict the overall survival of AIF in TCGA database. (e) PAK5 and AIF expression in 122 clinical breast tissue pairs. Lysates of tumor tissues (T) and matched adjacent noncancerous tissues (N) were analyzed using Western blotting. Twelve representative pairs are shown. (f) The indicated protein levels in (e) were statistically analyzed (****p < 0.001). (g) Spearman's rank test was used to analyze the correlation between AIF relative expression and PAK5 relative expression in 122 subjects. (h) Representative images of immunohistochemical staining showing PAK5 and AIF protein expression in breast cancer. Original magnification, 400×. IHC staining was scored, and a Pearson correlation test was performed. Note that the scores of some samples overlap. (i-l) Kaplan-Meier survival analysis (GraphPad) of the relationship between overall survival (upper) and disease-free survival (lower) in breast cancer cases and PAK5 and/or AIF expression. The subjects were divided into different groups based on the indicated PAK5 and AIF expression scores in the tumors: PAK5 low (n = 35) and PAK5 high (n = 40, i); AIF low (n = 36) and AIF high (n = 39, j). n = 15 for PAK5 low/AIF low, n=4 for PAK5 high/AIF low, n=12 for PAK5 low/AIF high, n = 44 for PAK5 high/AIF high; n = 35 for high nuclear translocation ratio of AIF, n = 40 for low nuclear translocation ratio of AIF.

To explore the role of PAK5 in the development of breast cancer, we overexpressed PAK5 in breast cancer cells and screened out the related proteins regulated by PAK5 by quantitative protein mass spectrometry. One of the proteins that attracted our attention and had significant differences in regulation is AIF (E9PMA0) (Fig. 1a). First, we predicted the expression of AIF in breast cancer and adjacent cancer in the TCGA database and found that AIF is highly expressed in breast cancer (Fig. 1b). At the same time, the overall survival of AIF was analyzed in GSE31519 (Fig. 1c) and TCGA databases (Fig. 1d). Patients with high AIF expression had a poor prognosis. To further verify the correlation between AIF and PAK5, we detected 122 pairs of fresh frozen clinical breast cancer tissues and paired adjacent nonneoplastic tissues, and found that the protein expression of PAK5 and AIF in breast cancer was higher than that in para cancer (Figs. 1e and 1f). As shown, expression of AIF and PAK5 showed a remarkable correlation (r = 0.512; P<0.0001, Spearman's rank correlation test) (Fig. 1g). Meanwhile, we found that PAK5 and AIF had cross-talk at protein level in breast cancer cells (Fig.S1 and S2). Besides, immunohistochemical staining and histopathological analysis of 75 cases of breast cancer showed that PAK5 was positively correlated with AIF expression (r = 0.522; P<0.001) (Fig. 1h). Kaplan-Meier survival analysis showed that high PAK5 and AIF levels were associated with poor overall survival (Figs. 1i and 1j, upper) and poor disease-free survival (Figs. 1i and 1j, lower). In addition, we compared the correlation between the expression levels of PAK5 and AIF and tumor stage in supplement table 1. The results showed that the expression of PAK5 and AIF was positively correlated with tumor stage in 75 cases of breast cancer. Stratification of patients based on PAK5 and AIF levels and AIF nuclear translocation ratio further improved the predictive capability (Figs. 1k and 1l).

### PAK5 directly binds to AIF

**Figure 2:**
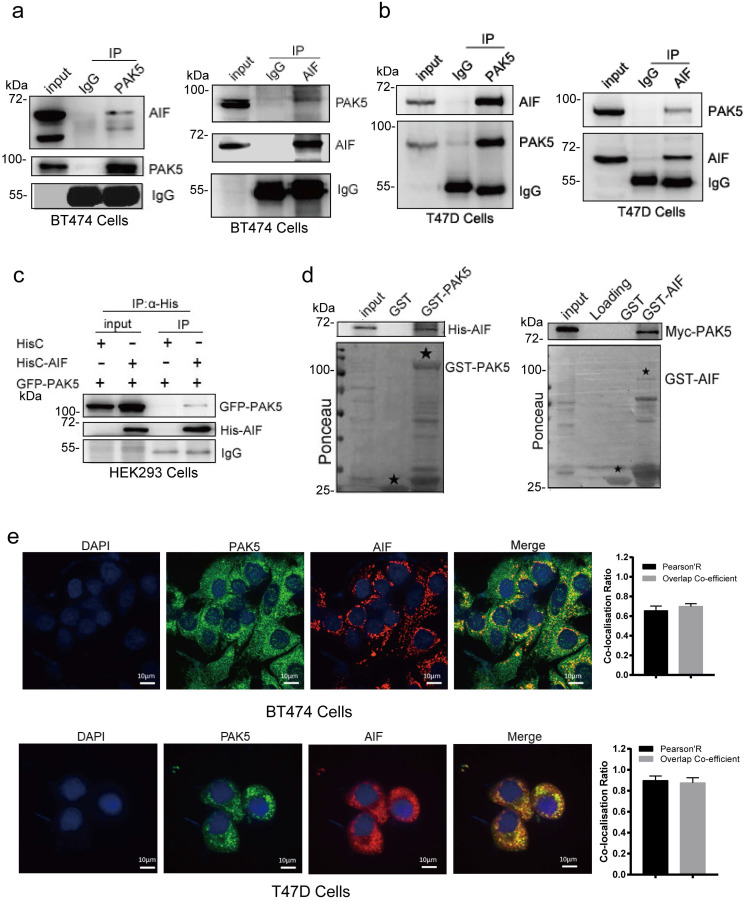
**PAK5 directly binds to AIF.** (a,b) Endogenous AIF interacts with PAK5. BT474 cells/T47D lysates were immunoprecipitated with the anti-PAK5, anti-AIF or IgG. Precipitates were analyzed by western blot. (c) Exogenous PAK5 interacts with AIF. Total lysates were subjected to immunoprecipitation and western blot. (d) PAK5 directly binds to GST-AIF *in vitro*. Black stars indicate GST and GST-fusion proteins. (e) Co-localization of PAK5 and AIF. Nucleus was stained with DAPI (4', 6 diamidino-2-phenylindole). Yellow indicates co-localization. Original magnification, ×600. The Pearson's correlation and overlap co-efficient were shown in bar graph format (30 cells) from three independent experiments were analyzed (error bars, SEM).

To verify the interaction between PAK5 and AIF, we performed a coimmunoprecipitation (Co-IP) assay to validate the PAK5-AIF interaction. The endogenous interaction of PAK5 with AIF was demonstrated in BT474 and T47D breast cancer cells (Fig. 2a and Fig. 2b). Moreover, the interaction between GFP-tagged PAK5 and His-tagged AIF was observed in HEK293 cells (Fig. 2c). In addition, a GST pull-down assay showed that PAK5 and AIF directly interacted *in vitro* (Fig. 2d). To further characterize the interaction between PAK5 and AIF, we examined the co-localization of endogenous AIF with PAK5 in BT474 and T47D cells. It was observed that AIF co-localized with PAK5 at the cytoplasm (Fig. 2e). Taken together, our findings suggest that AIF is a PAK5-binding protein in breast cancer cells.

### PAK5 inhibits AIF release by regulating mitochondrial membrane permeability and potential

**Figure 3:**
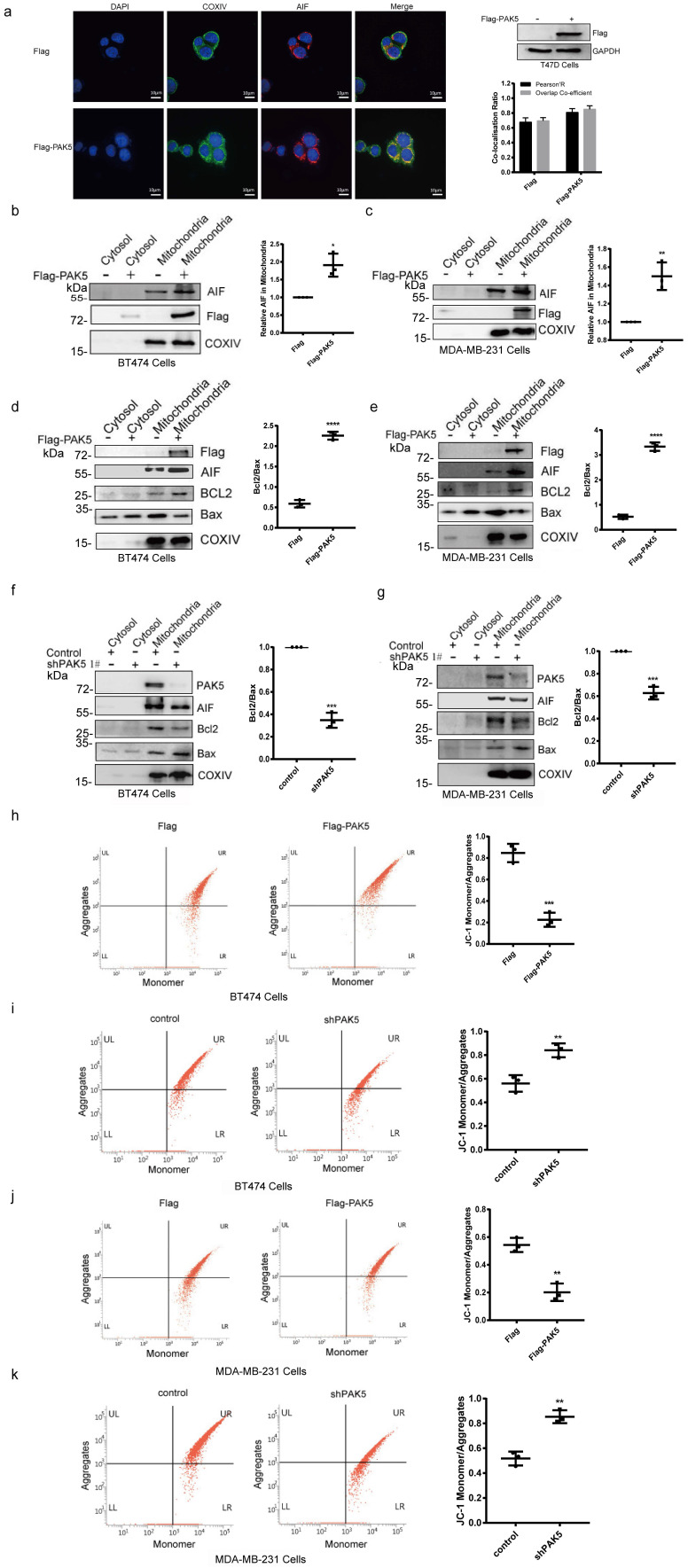
** PAK5 inhibits AIF release by regulating mitochondrial membrane permeability and potential.** (a) PAK5 could increase the co-localization of AIF and COXIV (mitochondrial protein). Nucleus was stained with DAPI (4', 6 diamidino-2-phenylindole). Yellow indicates co-localization. Original magnification, × 600. The Pearson's correlation and overlap co-efficient were shown in bar graph format (30 cells) from three independent experiments were analyzed (error bars, SEM). (b,c) PAK5 could increase the expression of AIF in mitochondria. Cells were stably transfected with Flag-tagged PAK5 and extracted mitochondrial protein. Protein expression levels were determined by Western blotting. The data are shown as the mean _ SEM of triplicate experiments (*p < 0.05, **P < 0.01 vs. Control, n =3). (d-g) PAK5 could reduce membrane permeability of mitochondria. Cells were stably transfected with Flag-tagged PAK5 (d,e) or infected with PAK5-RNAi lentivirus (f,g) and extracted mitochondrial protein. Protein expression levels were determined by Western blotting. The data are shown as the mean _ SEM of triplicate experiments (***p < 0.001, ****p < 0.0001 vs. Control, n =3). (h-k) PAK5 can increase the membrane potential of mitochondria. Cells were stably transfected with Flag-tagged PAK5 (h,j) or infected with PAK5-RNAi lentivirus (i,k), the change in ΔΨm was examined using JC-1 staining assay. The ratio of fluorescent intensity of J-aggregates and monomers in treated cells is shown in e and f. The data are shown as the mean _ SEM of triplicate experiments (**P < 0.01, ***p < 0.001 vs. Control, n =3).

AIF needs to be cleaved into 57 kDa soluble apoptotic protein in mitochondria to perform its apoptotic function, and AIF translocation through the mitochondrial membrane to the cytosol and to the nucleus is a crucial initiating step in apoptosis. The release of apoptosis factors in mitochondria is due to the loss of mitochondrial function, which is closely related to the increase of mitochondrial membrane permeability and depolarization of membrane potential. In Fig. S4 and 3a, we found that PAK5 and AIF could be localized in mitochondria, and overexpression of PAK5 increased the co-localization of AIF and COXIV protein which is a mitochondria marker. In addition, we overexpressed PAK5 in breast cancer cells and found the expression of AIF in mitochondria was increased (Fig. 3b and 3c). It has been reported that the release of AIF from mitochondria is related to the expression of BCL2 and Bax 23. We measured the expression levels of BCL2 and Bax in mitochondria to evaluate the permeability of the mitochondrial membrane. The results showed that the ratio of BCL2/Bax was increased when PAK5 overexpressed (Figs. 3d and 3e), while PAK5 knockdown led to opposing effects (Figs. 3f and 3g), indicating that PAK5 decreased mitochondrial membrane permeability. JC-1 staining and flow cytometry was used to detect the changes of mitochondrial membrane potential. Accordingly, using cells overexpressing PAK5, we verified that the mitochondrial membrane potential was increased, while PAK5 knockdown led to opposing effects (Figs. 3h-3k). These results suggest that PAK5 on mitochondria inhibits AIF release by decreasing mitochondrial membrane permeability and increasing membrane potential.

### PAK5 inhibits apoptosis by blocking AIF nuclear translocation

**Figure 4:**
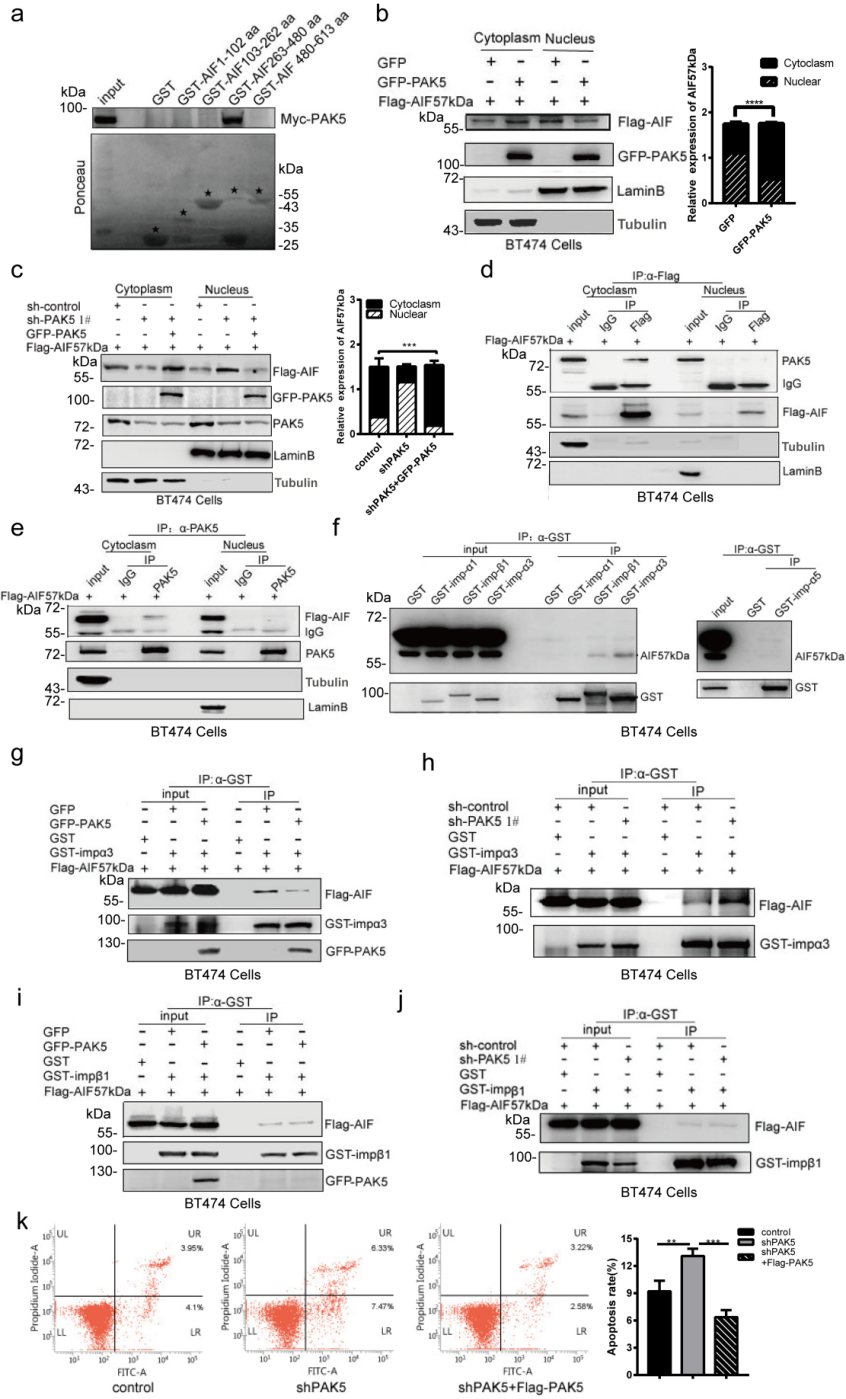
** PAK5 inhibits apoptosis by blocking AIF nuclear translocation.** (a) PAK5 directly binds to AIF amino acids 263 and 480. Black stars indicate GST and GST-fusion proteins. (b,c) Separation of nucleus and cytoplasm experiment showing that PAK5 expression decreased the nuclear translocation of AIF. The data are shown as the mean _ SEM of triplicate experiments (***p < 0.001, ****p < 0.0001 vs. Control, n =3). (d,e) PAK5 and AIF interact in the cytoplasm. Separation of cytoplasmic protein and nuclear protein with nucleoplasm separation Kit, lysates were subjected to immunoprecipitation and western blot with antibodies as indicated. (f) The interaction of AIF with importins was determined by immunoprecipitation assay. Lysates were immunoprecipitated with anti-GST antibody and immunoblotted with indicated antibodies. (g) Overexpression of PAK5 diminished AIF interaction with importin α3. Lysates were immunoprecipitated with anti-GST antibody and immunoblotted with indicated antibodies. (h) Knockdown of PAK5 enhanced AIF interaction with importin α3. Lysates were immunoprecipitated with anti-GST antibody and immunoblotted with indicated antibodies. (i,j) Overexpression(i)/knockdown(j) of PAK5 had no effect on AIF interaction with importin α3. Lysates were immunoprecipitated with anti-GST antibody and immunoblotted with indicated antibodies. (k) PAK5 can inhibit apoptosis of breast cancer cells.BT474 cells transfected with the indicated constructs. The apoptosis was detected by flow cytometry. On the two-dimensional scatter diagram, PI and annexin V are x and Y axes respectively, the second quadrant represents apoptotic cells, and the fourth quadrant represents early apoptotic cells. The data are presented as a histogram of the mean _ SEM of three independent experiments (**p < 0.01, ***p < 0.001, n =3).

To explore the effect of PAK5 on the function of AIF, we designed a truncated plasmid according to the functional domain of AIF. We identified the specific binding region of AIF and PAK5 by GST pull-down method. We found that PAK5 was bound with 263-480aa of AIF (Fig. 4a), including the nuclear localization signal of AIF. This suggests that PAK5 may affect the process of AIF entering the nucleus. To confirm the effect of PAK5 on AIF nuclear translocation, we overexpressed PAK5 and found that nuclear translocation of AIF was decreased significantly (Fig. 4b). PAK5 knockdown resulted in increased nuclear translocation of AIF, meanwhile PAK5 overexpression reversed the effect on AIF nuclear translocation (Fig. 4c). Next, we overexpressed PAK5 and AIF57kDa in breast cancer cells and verified the interaction between PAK5 and AIF in the cytoplasm by immunoprecipitation (Figs. 4d and 4e). Previous experiments showed that PAK5 could phosphorylate E47 and promote the phosphorylated E47 to introduce into the nucleus in an importin α3/α5 dependent manner 24. We tested the interaction between AIF and importins and clearly showed that AIF interacts with importin α3 or β1 (Fig. 4f). To further clarify the potential effect of PAK5 on AIF introduction, we detected the interaction between AIF and importin α3 and importin β1 in the presence or absence of PAK5. The results showed that AIF interacted more strongly with importin α3 without PAK5 (Figs. 4g, 4h, and S6), but had little effect on the interaction between AIF and importin β1 (Figs. 4i, 4j and S6). These results indicate that PAK5 could prevent AIF from introducing into nuclear in an importin α3 dependent manner. Because AIF entering the nucleus was a common event of cell caspase-independent apoptosis, we knocked down PAK5 in breast cancer cells, and found that apoptosis was increased; meanwhile PAK5 overexpression reversed the effect on apoptosis (Fig. 4k). In Fig. S5, cleaved PARP is an early indicator of apoptosis. When we knocked down PAK5, cleaved PARP was increased significantly. After adding caspase inhibitor, knocking down PAK5 can still activate PARP and induce caspase- independent apoptosis. These results suggested that PAK5 can inhibit AIF from entering the nucleus and prevent caspase- independent apoptosis.

### PAK5 inhibits AIF nuclear translocation by phosphorylation of AIF on Thr281

**Figure 5:**
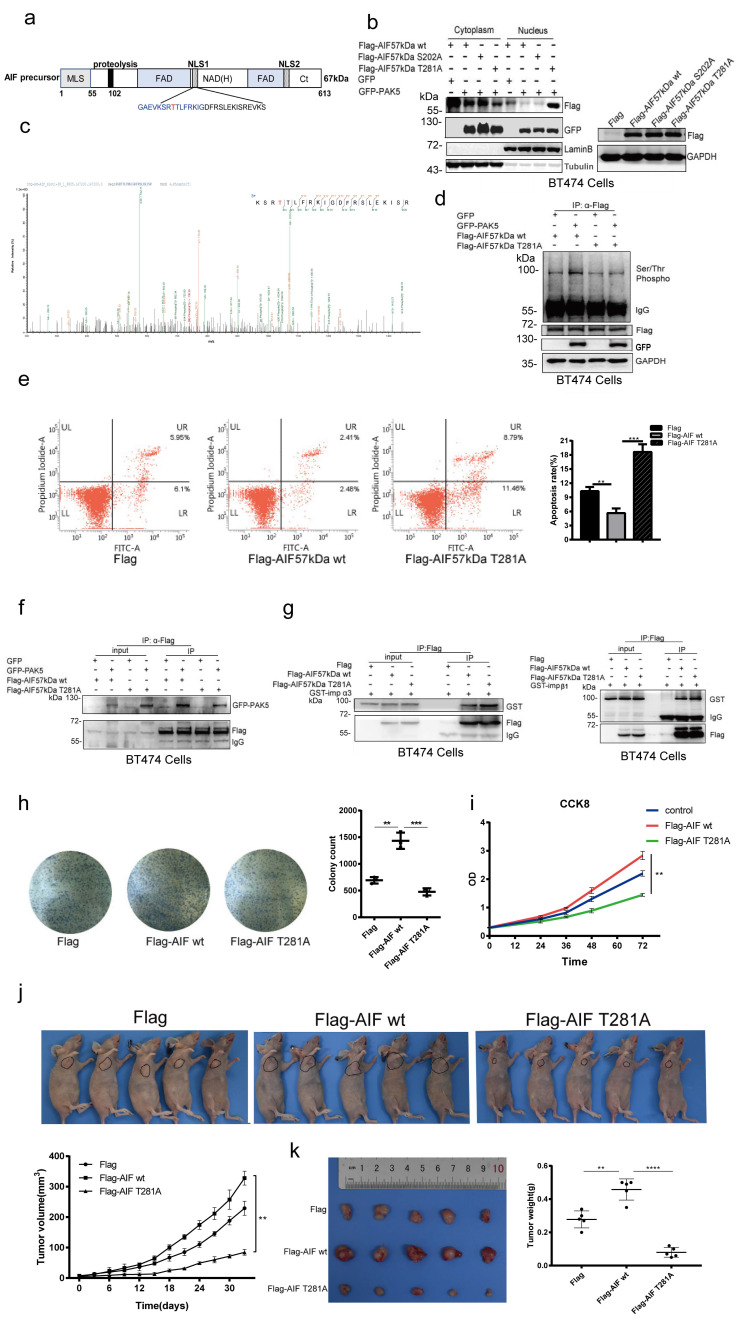
** PAK5 inhibits AIF nuclear translocation by phosphorylation of AIF on Thr281.** (a) Naturally occurring transcripts corresponding to the AIF precursor (b) AIF phosphorylation leads to decrease of nuclear translocation. Collection of cells for nuclear and cytoplasmic separation and western blot analysis. On the right side is the total protein extracted from the same batch of transfected cells, and the transfection efficiency of plasmids is detected by Western blot. (c) Analysis of phosphorylation sites and peptides of AIF by PAK5 by mass spectrometry. (d) PAK5 phosphorylated the T281 site of AIF. Lysates were immunoprecipitated with anti-Flag antibody and immunoblotted with indicated antibodies. (e) AIF T281A mutation can increase apoptosis. The apoptosis was detected by flow cytometry. The data are presented as a histogram of the mean _ SEM of three independent experiments (**p < 0.01, ***p < 0.001, n =3). The transfection efficiency of the plasmid was detected by Western blot (Fig.S8). (f) AIF T281A mutation and PAK5 interaction was weakened than AIF WT. Lysates were immunoprecipitated with anti-Flag antibody and immunoblotted with indicated antibodies. (g) AIF T281A mutation and importin α3 interaction was enhanced than AIF WT without affecting importinβ1. Lysates were immunoprecipitated with anti-Flag antibody and immunoblotted with indicated antibodies. (h)AIF phosphorylation promoted colony formation. Colonies of BT474 cells stably expressing control, AIF WT or AIF T281A were monitored. Representative pictures of the colonies are shown. The data are presented as a histogram of the mean _ SEM of three independent experiments (**p < 0.01, ***p < 0.001, n =3). (i) CCK8 was added at 0, 24, 36, 48, 72 hours of cell attachment in BT474 Cell, incubated at 37 ° for 2 hours, and detected by enzyme labeling instrument. The data are presented as a histogram of the mean _ SEM of three independent experiments (**p < 0.01, n =3). (j) BT474 cells (2× 10^6^ cells) stably expressing vector control, AIF WT or AIF T281A were injected subcutaneously into the right flank of nude mice. Mice were imaged at 30 days after injection. Tumor diameter was measured at the indicated time points, and tumor volume was calculated. The results are presented as the mean SEM of 5 mice per group per time point (****p < 0.0001). (k) Tumors formed by cells described in (i) were extracted from mice and photographed. Tumor weight in mice from this experiment was measured upon autopsy at Day 30, and the results are presented as a histogram (**p < 0.01, ***p < 0.001).

Since PAK5 is a well-known serine/threonine protein kinase, we next attempt to determine whether PAK5 phosphorylates AIF. According to GPS software prediction and bioinformatics analysis, Ser-202 and Thr-281 were the two phosphorylation sites with the higher score (Fig. S7), and Thr-281 was included in the nuclear location signal of AIF (Fig. 5a). Next, we explored the possibility of PAK5 mediated phosphorylation in regulating the nuclear translocation of AIF. We found that PAK5 induced nuclear translocation of T281A mutant protein (AIF 57kDa T281A, nonphosphorylatable) rather than wild-type AIF (AIF 57kDa WT) and S202A mutant protein (AIF 57kDa S202A, nonphosphorylatable) (Fig. 5b). To confirm that AIF was a candidate for PAK5 phosphorylation, we detected the phosphorylation sites of AIF by PAK5 via protein mass spectrometry, including the T281 site of AIF (Fig. 5c). Therefore, we selected Thr-281 as the main phosphorylation site for further study. The classical phosphorylation motif of the group II PAK kinases is RKS/KRS 25, 26. We used the serine/threonine phosphorylation antibody of this motif to further detect PAK5 mediated AIF phosphorylation. The results showed that the phosphorylation level of wild-type AIF in cytosol but not AIF T281A increased with overexpression of PAK5 (Figs. 5d), indicating that PAK5 interacted with AIF and phosphorylated at T281 under physiological conditions. Then we overexpressed the wild-type (WT) and mutant (T281A) of AIF in breast cancer cells. Through apoptosis detection, we found that AIF-T281A was more likely to induce apoptosis than wild-type (Figs. 5e and S8). In order to elucidate the effect of AIF phosphorylation on its interaction with importin α3 or PAK5, we detected that AIF-T281A had weaker binding to PAK5 than the same amount of wild-type AIF (Fig. 5f), while the binding to importin α3 was enhanced without affecting its binding to importin β1 (Fig. 5g). These results suggested that phosphorylation of AIF-T281 was a necessary condition to prevent AIF from entering the nucleus. To determine the biological function of AIF phosphorylation, we evaluated the proliferation characteristics of cancer cells. Colony formation experiments showed that the number of colonies formed by AIF-WT stably expressed cells was significantly higher than that of control cells, while the expression of AIF-TA was slightly lower than that of control cells (Fig. 5h). Similarly, in the CCK8 experiment, it was found that the proliferation of AIF wild-type was faster than that of the control, while the proliferation of the mutant was slightly slower than that of the control (Fig. 5i). Next, *in vivo* evidence supporting the association between AIF phosphorylation and breast cancer progression was obtained from tumor xenograft studies. Cells expressing AIF-WT grew xenografts at a significantly faster rate than those in the AIF-TA and vector groups (Figs. 5j and 5k). These results suggested that PAK5 inhibited AIF from entering the nucleus through phosphorylation of AIF T281 site, thus inhibiting cell apoptosis.

### Expression levels of AIF in nucleus and cytoplasm reveal the clinical relevance between PAK5 and AIF nuclear translocation in breast cancer

**Figure 6:**
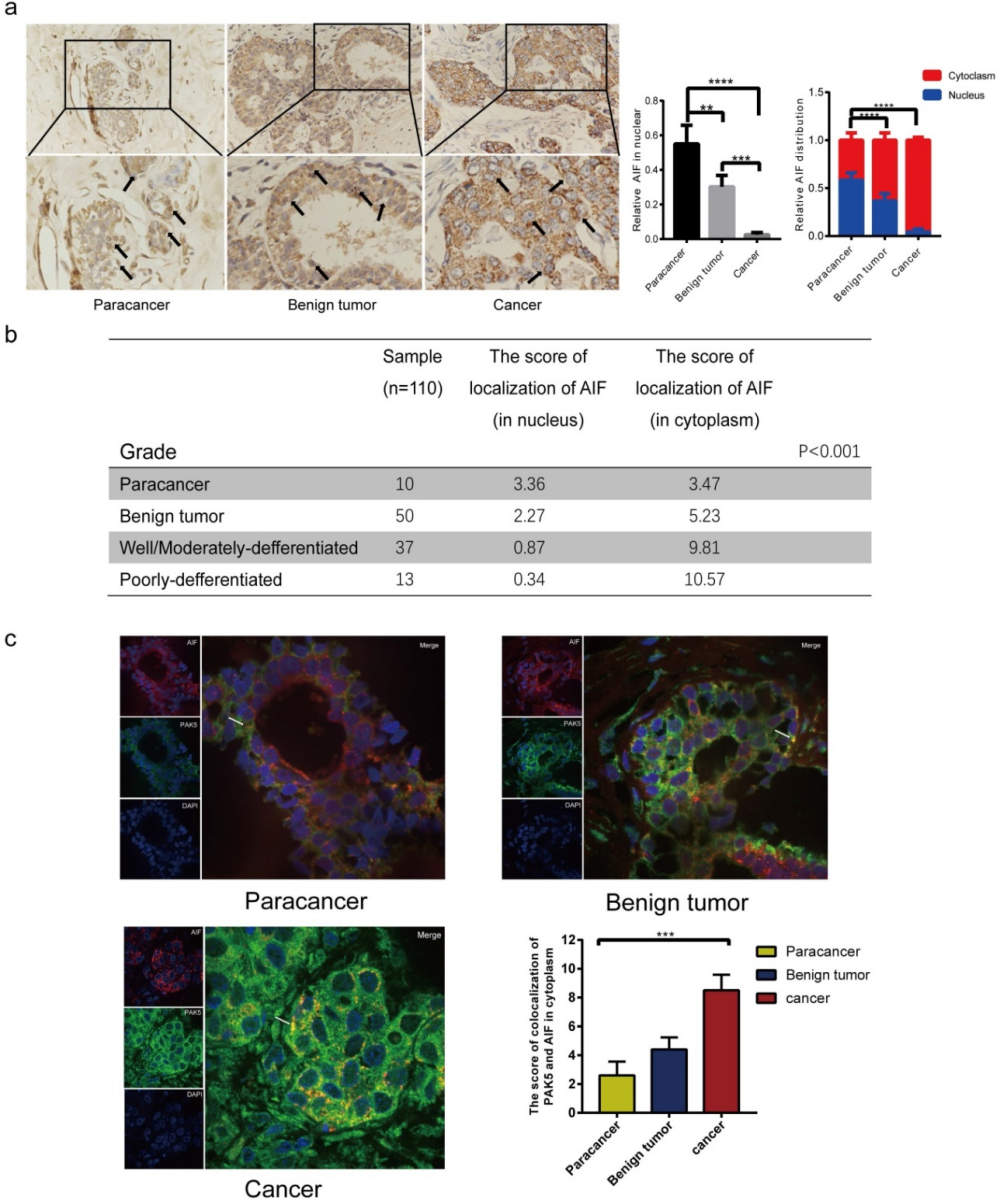
** Expression levels of AIF in nucleus and cytoplasm reveal the clinical relevance between PAK5 and AIF nuclear translocation in breast cancer.** (a) AIF expression was detected by immunohistochemical (IHC) staining. 10 cases of Para cancer, 50 cases of breast benign tumor and 50 cases of breast cancer were collected for immunohistochemical analysis. The percentage of AIF in total visual field cells with nuclear translocation and the percentage of AIF in nucleus or cytoplasm of cells with nuclear translocation were calculated from 10 independent visual fields (**P < 0.01, ***P < 0.001, ****P < 0.0001). (b) The nuclear translocation of AIF was negatively correlated with the malignant degree of tumor. The localization fraction of AIF in the nucleus was calculated and determined according to the intensity of staining cells (0-3) and the number of stained cells (0-4). The total score (0-12) was obtained. The black arrow in the figure indicates that the expression of AIF in the nucleus (***P < 0.001). (c) 10 cases of normal breast tissue, 10 cases of benign breast tumor, 10 cases of breast cancer were collected for immunofluorescence analysis. The specimens were fixed and incubated with anti PAK5 antibody (green), anti AIF antibody (red). The nuclei were stained with DAPI (blue). The co localization of PAK5 and AIF in the cytoplasm was calculated from 10 independent visual fields, and was determined according to the intensity of staining cells (0-3) and the percentage of stained cells (0-4). The total score (0-12) is obtained by multiplying the dyeing intensity with the fraction. The white arrow in the figure indicates that AIF and PAK5 are co-located in the cytoplasm (***P < 0.001).

We seek to further support our findings in human breast cancer. Under physiological conditions, apoptosis plays a vital role in maintaining the stability of the body environment. We compared the location and expression of AIF in paracancerous tissues, benign tumor and breast cancer. The results showed that nuclear translocation of AIF was apparent in paracancerous tissues and benign tumors (Fig. 6a), while in breast cancer tissues, the expression of AIF in cytoplasm was significantly increased and nuclear translocation was reduced. Histological grade analysis further confirmed that the nuclear translocation of AIF was negatively correlated with the malignant degree of tumor (P < 0.001) (Fig. 6b). To further confirm the clinical correlation between PAK5 and AIF, we studied the correlation between PAK5 expression and AIF translocation in breast cancer. The results showed that PAK5 was highly expressed in breast cancer, and the co-localization of PAK5 and AIF in the cytoplasm was significantly increased and the expression of AIF in nucleus was clearly decreased, indicating that the expression of PAK5 was negatively correlated with the nuclear translocation of AIF (Fig. 6c). Therefore, it provides strong support for our conclusion.

In conclusion, our results indicate that PAK5-mediated AIF phosphorylation can prevent AIF from entering the nucleus to inhibit cell apoptosis, thus promoting the occurrence and development of breast cancer.

## Discussion

**Figure 7:**
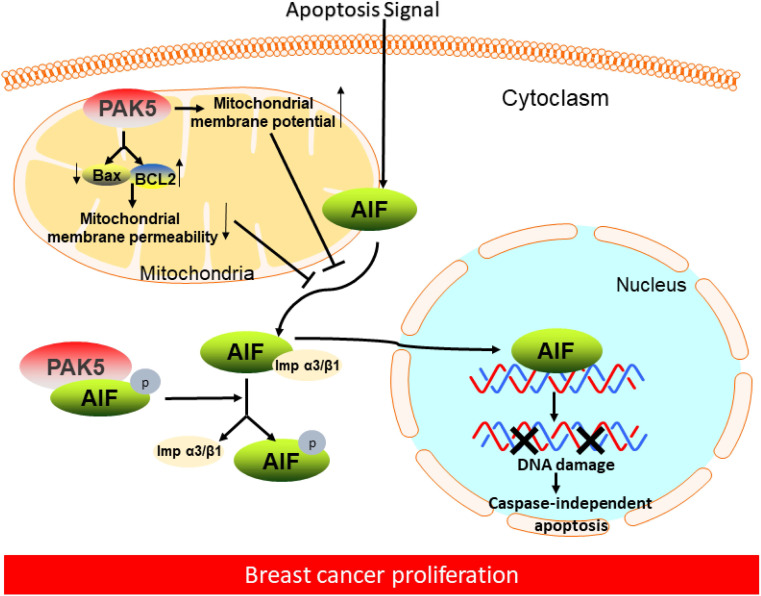
** Proposed model of PAK5-AIF signaling pathway in breast cancer proliferation.** In breast cancer cells, on the one hand, PAK5 prevents AIF from releasing from mitochondria by changing mitochondrial membrane permeability and membrane potential; on the other hand, PAK5 prevents AIF entering the nucleus through the phosphorylation of AIF in cytoplasm, thus inhibiting cell apoptosis.

In this study, we demonstrated that PAK5 could prevent AIF release from mitochondria by decreasing mitochondrial membrane permeability and increasing membrane potential. PAK5 could also phosphorylate AIF in cytoplasm. The interaction between phosphorylated AIF and importin α3 is weakened, thus preventing AIF from entering the nucleus in an importin α3-dependent manner, which was associated with histologic differentiation in breast cancer (Fig. 7). These results could help us better understand the molecular mechanism of PAK5 in promoting the occurrence and development of breast cancer.

As a common carcinogenic protein, PAK5 is a crucial regulator of breast cancer progression 10-12, but its potential molecular mechanism is still unclear. In this study, we investigated the mechanism of PAK5 inhibiting apoptosis by blocking the nuclear translocation of AIF. For the first time, we found a positive correlation between PAK5 and AIF expression in breast cancer. Survival analysis showed that patients with high expression of PAK5 and AIF had a poor prognosis. Besides, we found that AIF and PAK5 co-located in the cytoplasm rather than the nucleus of breast cancer. With the development of breast cancer, the co-localization of PAK5 and AIF in the cytoplasm increased significantly, while the nuclear translocation of AIF decreased. These data indicate that PAK5 plays a vital role in the nuclear translocation of AIF.

Mitochondria are the internal pathway of cell apoptosis, which determines cells survive 27. The intrinsic pathway involves the increase of mitochondrial outer membrane permeability, which leads to the release of various apoptosis factors from the membrane space into the cytoplasm, such as Smac/Diablo, endonuclease G and AIF 28. AIF is a flavoprotein existing in mitochondrial membrane space. It not only has the function of redox and electron transfer, but also can promote apoptosis, which plays an important role in maintaining the normal physiological activities of cells. Under the stimulation of exogenous death trigger signal, AIF was released from mitochondria to cytoplasm and then into nuclear, which induced caspase-independent apoptosis 15, 16. The release of AIF from mitochondria can be regulated by many factors. It has been reported that PAK5 is mainly located in mitochondria 29. PAK5 can regulate mitochondrial protein expression and mitochondrial distribution and play an anti-apoptotic role 30, 31. Mitochondrial apoptotic factors can be released through the outer membrane pores formed by Pro-apoptotic Bcl-2 family members, and they are in a latent state in healthy cells. They undergo conformational changes and homologous and heterologous oligomerization, and form channels in the outer membrane of mitochondria. They can initiate mitochondrial death and promote AIF translocation by increasing mitochondrion division and changing ridge formation 15, 32. In addition, the expression of Bcl-2 can cause MPTP pore closure by isolating Bax and Bak in order to block the release of AIF from mitochondria and inhibit apoptosis 23. These results suggest that Bcl-2 family proteins are involved in the release of AIF from mitochondria. Besides, the opening of PTP is also regulated by mitochondrial membrane potential. The dissipation of mitochondrial membrane potential is related to the cytoplasmic translocation of AIF 33-35. In the report, we found that PAK5 could increase the ratio of BCL2/Bax and mitochondrial membrane potential, and inhibit the release of AIF by reducing the permeability of mitochondrial membrane.

We show that the entry of AIF into nucleus also depends on importin α3/β1, and the interaction between AIF and importin α3 is regulated by PAK5. PAK5 can significantly reduce the nuclear retention rate of AIF and the interaction between AIF and importin α3, thus inhibiting cell apoptosis. We confirmed that this regulation is related to the phosphorylation of AIF by PAK5. According to the literature reports, human AIF contains two NLS motifs: NLS1 (amino acid 278-302) and NLS2 (amino acid 445-451) 17. Our results show that PAK5 can phosphorylate Thr-281 of AIF, which is included in its NLS1 sequence. Thr-281 phosphorylation of AIF can inhibit its entry into the nucleus through importin α3, thus inhibiting apoptosis.

The new model we described shows how PAK5 can prevent apoptosis in breast cancer cells by inhibiting AIF translocation. It is emphasized that apoptosis is necessary to maintain the normal physiological state of cells 36-38. Interestingly, we found that the expression of AIF in breast cancer was higher than that in benign breast disease and paracancerous tissue, but it was mostly in the cytoplasm and rarely expressed in the nucleus. The analysis of clinical results showed that the increase of AIF nuclear translocation was negatively correlated with the high degree of malignancy. In addition, we confirmed that the nuclear translocation of AIF was decreased and the co-localization of PAK5 and AIF in the cytoplasm was increased with the increase of tumor malignancy, which indicated that PAK5 prevented cell apoptosis by inhibiting AIF from entering the nucleus, thus promoting the development of breast cancer.

In conclusion, PAK5 can reduce AIF nuclear translocation by changing mitochondrial function and phosphorylation of AIF, thus promoting the development of breast cancer. These results provide clues to reveal the molecular mechanism of promoting breast cancer cell proliferation, and also provide useful enlightenment for breast cancer treatment strategies.

## Supplementary Material

Supplementary figures and tables.
