# Supplementary material for: PAK5-mediated AIF phosphorylation inhibits its nuclear translocation and promotes breast cancer tumorigenesis
Source: Int J Biol Sci. 2021 Mar 27;17(5):1315–27. doi: 10.7150/ijbs.58102 (PMC8040471; doi:10.7150/ijbs.58102)
Supplement: Supplementary file 1 — Supplementary figures and tables. [file ijbsv17p1315s1.pdf]

Supplement table 1: Comparison of expression levels of PAK5 and AIF with tumor stage in breast cancer samples.

	**Tumor stage**		
**Factor**	**I (n=25)**	**II/III (n=50)**	**P value**
PAK5			**0.011***
Low	14	13	
High	11	37	
AIF			**0.002****
Low	18	17	
High	7	33	

*Indicated statistical significance (P< 0.05).

**Indicated statistical significance (P< 0.01).

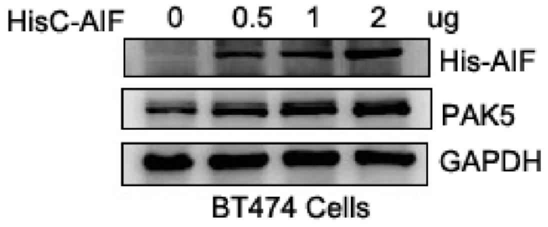

Supplement figure 1: Overexpression of AIF upregulates PAK5. BT474 cells transiently expressing different doses of HisC-AIF were analyzed with the indicated antibodies via Western blotting.

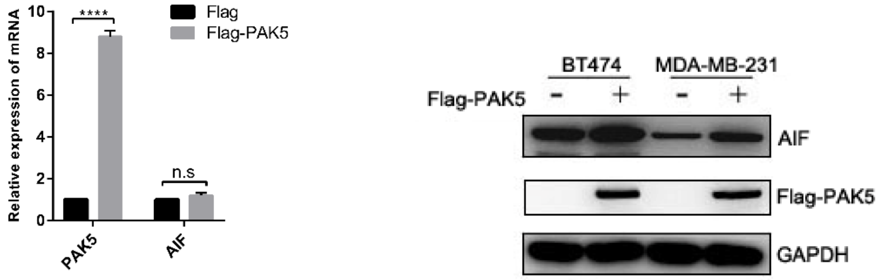

Supplement figure 2: Overexpression of PAK5 upregulates AIF at protein level. BT474 cells were stably overexpressed Flag-PAK5 and analyzed AIF expression at transcription level(left). BT474 cells and MDA-MB-231 cells were stably overexpressed Flag-PAK5 and analyzed with the indicated antibodies via Western blotting(right).

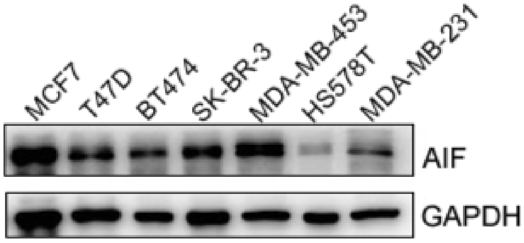

Supplement figure 3: Analysis of AIF protein expression level in breast cancer cell lines.

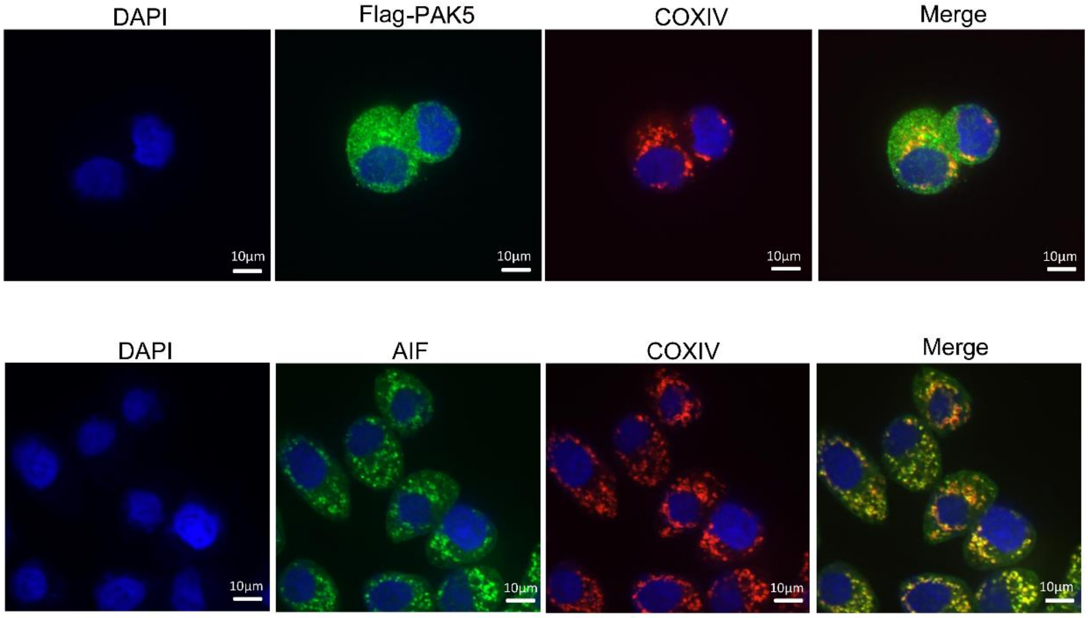

Supplement figure 4: Co-localization of Flag-PAK5, AIF and COXIV. COXIV is a mitochondrial protein. Alexa Fluor 488 (green) or Alexa Fluor 546 (red) was used to detect Flag-PAK5, AIF and COXIV respectively; nucleus was stained with DAPI (4', 6 diamidino-2-phenylindole). Yellow indicates co-localization. Original magnification, × 600.

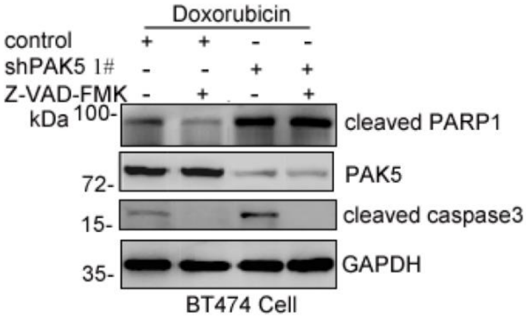

Supplement figure 5: BT474 cells transfected with the indicated constructs were exposed to 0.5μM Doxorubicin and 10μM Z-VAD-FMK for 12 hr in culture medium. Western

blotting was performed with the indicated antibodies.

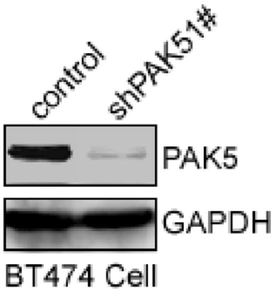

Supplement figure 6:BT474 cells infected with PAK5-RNAi lentivirus. PAK5 protein expression in the cells was detected by western blot. GAPDH served as protein loading control.

Predicted Sites

Position	Code	Kinase	Peptide	Score	Cutoff
209	S	STE	SIYFQPPSFYVSAQD	0.886	0.744
304	T	STE	SREVKSITIIGGGFL	0.789	0.744
391	T	STE	KDGRKVETDHIVAAV	1.25	0.744
480	S	STE	AKPYWHQSMFWSDLG	0.991	0.744
526	T	STE	SATEQSGTGIRSESE	1.259	0.744
532	S	STE	GTGIRSESETESEAS	0.855	0.744
547	T	STE	EITIPPSTPAVPQAP	0.899	0.744
249	S	STE/STE20	MVKLNDGSQITYEKC	1.198	1.173
281	T	STE/STE20	GAEVKSRTTLFRKIG	2.203	1.173
521	T	STE/STE20	QDNPKSATEQSGTGI	1.581	1.173
202	S	STE/STE20/PAKB	QWNGKERSIYFQPPS	6.3	3.44
268	S	STE/STE20/PAKB	GGTPRSLSAIDRAGA	3.9	3.44
292	S	STE/STE20/PAKB	RKIGDFRSLEKISRE	4.8	3.44
★ 202	S	STE/STE20/PAKB/PAK5	QWNGKERSIYFQPPS	10	4.45
209	S	STE/STE20/PAKB/PAK5	SIYFQPPSFYVSAQD	4.5	4.45
266	S	STE/STE20/PAKB/PAK5	ATGGTPRSLSAIDRA	6	4.45
★ 281	T	STE/STE20/PAKB/PAK5	GAEVKSRTTLFRKIG	7.25	4.45
292	S	STE/STE20/PAKB/PAK5	RKIGDFRSLEKISRE	4.5	4.45

Supplement figure 7: GPS software predicted the phosphorylation sites of AIF by PAK5.

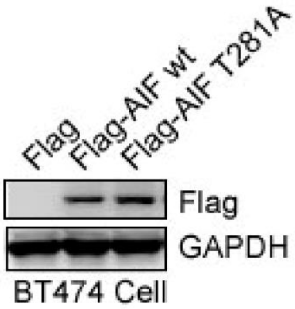

Supplement figure 8: BT474 cells transfected with the indicated constructs and the transfection efficiency of the plasmid was detected by Western blot.
